# Adapting to Change: Care Partner Challenges and Strategies in Mild Cognitive Impairment and Mild Dementia

**DOI:** 10.1093/geroni/igaf122.796

**Published:** 2025-12-31

**Authors:** Cassandra Dictus, Youngmin Cho, Ya-Ning Chan, Chiao-Hsin Teng, Jing Wang, Victoria Crowder, Ruth Anderson, Bianca Shieu

**Affiliations:** Duke University, Durham, North Carolina, United States; Johns Hopkins University, Baltimore, Maryland, United States; Duke University, Durham, North Carolina, United States; Chang Gung University, Taoyuan City, Taoyuan, Taiwan (Republic of China); University of New Hampshire, Durham, New Hampshire, United States; The University of North Carolina at Chapel Hill, Chapel Hill, North Carolina, United States; Duke University, Durham, North Carolina, United States; University of Texas Health Science Center at San Antonio, San Antonio, Texas, United States

## Abstract

Care partners are critical for helping people living with mild cognitive impairment (MCI) and mild dementia (MD) maintain daily routines. This study compared the experiences of care partners working to support people living with MCI and MD with their daily tasks. Using a secondary qualitative analysis, we analyzed recorded intervention coaching sessions where care partners (10 MCI care partners and 8 MD care partners) learned communication techniques to support individuals with MCI and MD in learning oral hygiene techniques. Guided by the Adaptive Leadership Framework for Chronic Illness, we applied directed content analysis to explore patterns in their caregiving experiences. Findings revealed that care partners encountered a range of adaptive challenges, from managing functional changes and communication difficulties to balancing caregiving responsibilities with personal needs. MCI care partners expressed concerns about future cognitive decline, whereas MD care partners focused on balancing efficiency with autonomy in caregiving. Strategies varied by cognitive stage: MD care partners relied on tactile cues to reinforce routines, while MCI care partners were more collaborative and less directive in their communication. Care partners in both MCI and MD groups also engaged in adaptive work by refining techniques, seeking support, and adjusting their approaches over time. These findings underscore the importance of tailoring interventions and clinical support based on the stage of cognitive decline. Programs designed for care partners should address relationship dynamics, communication strategies, access to resources, and future planning to enhance caregiving experiences and improve outcomes for both care partners and individuals with MCI and MD.

